# Blood pressure control in Asia: from evidence to practice

**DOI:** 10.1016/j.lanwpc.2025.101705

**Published:** 2025-09-30

**Authors:** The Lancet Regional Health – Western Pacific

Proper blood pressure management is particularly pertinent in Asia, where the prevalence of hypertension is increasing and the absolute number of affected individuals is substantial, with an estimate of over 500 million people in 2019, which was nearly half of the global total. The impact of hypertension on cardiovascular diseases such as stroke, and on chronic kidney disease related to hypertension, tends to be greater among Asian populations compared with White people, and the management remains inadequate—with the controlled hypertension rate less than 20%.

A blood pressure target of 140/90 mm Hg is widely adopted as the standard for hypertension management following the JNC 7 Report in 2003. In 2015, the SPRINT trial introduced the concept of intensive blood pressure control, aiming for systolic blood pressure below 120 mm Hg. Since then, numerous studies and clinical trials have been conducted in the Asia–Pacific region to determine the optimal blood pressure target, such as the STEP study in China and the OPTIMAL-BP study in South Korea. The recent evidence from the first individual participant data meta-analysis of six landmark randomised controlled trials provides robust support for the benefit of intensive blood pressure control. This analysis quantified the benefit–harm trade-offs of intensive blood pressure targets (120 mm Hg or 130 mm Hg) compared with standard control (140 mm Hg), ultimately demonstrating an overall favourable benefit–harm profile of intensive blood pressure control.

In this meta-analysis, more than 80% of the participants were Asian, further supporting the potential benefits of intensive blood pressure control in the context of Asia. Despite the evidence on the benefits, there are knowledge–practice gaps on intensive blood pressure control in Asia between research evidence, clinical guidelines, and real-world practice.

First, patient subgroups should be carefully considered when applying the targeted blood pressure. Variability exists in adverse event reporting across trials with major or minor differences in participant characteristics, such as race, ethnicity, age, and comorbidities. Particularly, intensive blood pressure treatment-related adverse events such as hypotension, syncope, and kidney-related outcomes occurred more frequently in older (age ≥65 years) patients compared with younger patients. Therefore, different patient populations might need tailored approaches rather than uniform care in regard to the blood pressure target, treatment threshold, treatment duration, and the type of medication. Notably, the recent paper published in *The Lancet* develops an efficacy calculator to facilitate the selection of antihypertensive drug regimens including type, dosage, and combinations, based on pretreatment blood pressure and the desired blood pressure reduction. The 2025 ACC/AHA Hypertension Guideline recommends using the Predicting Risk of Cardiovascular Disease Events calculator for risk assessment when treating patients with hypertension. To optimise the benefits for different patient subgroups, individualising hypertension treatment strategies based on patient risk profiles are necessary.

The development of country-specific guidelines grounded in local evidence is needed. Unlike Europe and North America, which have established European and American guidelines, there is currently no region-specific hypertension management guideline for Asia. This raises the question of whether harmonising hypertension guidelines across Asia is necessary, or if country-specific, local, and national guidelines would be more relevant and effective. The latter approach might be more appropriate given the region's large diversity in epidemiology, cultural and lifestyle factors, health-care resources, and moreover, challenges related to hypertension management differ across countries. For example, substantial diagnosis gap, treatment inertia at the patient and system level, as well as high attrition rates during follow-up could be the primary barriers in low-income and middle-income countries (LMICs) or rural areas; while conversely, improving the suboptimal controlled rates despite high treatment rates might be a challenge for some high-income countries. Conducting more implementation studies to evaluate different aspects of real models for hypertension management, especially in resource-limited settings, as well as the development of practical translational protocols is essential. LMICs likely need simple, effective, and easily implementable protocols to manage most hypertension cases in community. A combination of accessible community health centres, straightforward protocols, and technology aids can be effective, provided they are tailored to the specific context of local health networks.

As the first step to implement intensive blood pressure control in clinical practice, governments are encouraged to develop and endorse national guidelines that emphasise intensive blood pressure control, supported by funding initiatives to improve medication affordability, access, and adherence. Local health ministries should also have a lifetime strategy to address blood pressure control including legislative tools. Health-care providers should be empowered with the knowledge and capacity on current guidelines and the adoption of intensive blood pressure control protocols through up-to-date training. Multidisciplinary teams could be established to facilitate continuous monitoring and assessment of potential adverse effects, allowing for timely adjustments to management strategies. The development and distribution of single–pill combinations could help to improve treatment compliance. The use of innovative technologies—such as digital health tools, artificial intelligence, and wearable devices—can improve measurement accuracy and address visit-to-visit blood pressure variability. Additionally, improving public awareness and health literacy, and establishing robust follow-up protocols and data management systems can support ongoing patient engagement.

The main barrier to blood pressure control in Asia is how to transform evidence and concepts into actionable clinical practice. Multisector efforts are essential to facilitate the integration of blood pressure control strategies into routine clinical practice and eventually translate to improved patient outcomes.

